# A Metabolomic Approach in Search of Neurobiomarkers of Perinatal Asphyxia: A Review of the Current Literature

**DOI:** 10.3389/fped.2021.674585

**Published:** 2021-06-25

**Authors:** Marie Julie Debuf, Katherine Carkeek, Fiammetta Piersigilli

**Affiliations:** Division of Neonatology, Cliniques Universitaires Saint Luc, Université Catholique de Louvain, Bruxelles, Belgium

**Keywords:** neonate, perinatal asphyxia, hypoxic-ischemic encephalopathy, neurobiomarkers, metabolomics

## Abstract

Perinatal asphyxia and the possible sequelae of hypoxic-ischemic encephalopathy (HIE), are associated with high morbidity and mortality rates. The use of therapeutic hypothermia (TH) commencing within the first 6 h of life—currently the only treatment validated for the management of HIE—has been proven to reduce the mortality rate and disability seen at follow up at 18 months. Although there have been attempts to identify neurobiomarkers assessing the severity levels in HIE; none have been validated in clinical use to date, and the lack thereof limits the optimal treatment for these vulnerable infants. Metabolomics is a promising field of the “omics technologies” that may: identify neurobiomarkers, help improve diagnosis, identify patients prone to developing HIE, and potentially improve targeted neuroprotection interventions. This review focuses on the current evidence of metabolomics, a novel tool which may prove to be a useful in the diagnosis, management and treatment options for this multifactorial complex disease. Some of the most promising metabolites analyzed are the group of acylcarnitines: Hydroxybutyrylcarnitine (Malonylcarnitine) [C3-DC (C4-OH)], Tetradecanoylcarnitine [C14], L-Palmitoylcarnitine [C16], Hexadecenoylcarnitine [C16:1], Stearoylcarnitine [C18], and Oleoylcarnitine [C18:1]. A metabolomic “fingerprint” or “index,” made up of 4 metabolites (succinate × glycerol/(β-hydroxybutyrate × O-phosphocholine)), seems promising in identifying neonates at risk of developing severe HIE.

## Introduction

Perinatal asphyxia is defined as the deprivation of oxygen occurring around the time of birth, and despite improved antenatal and peripartum care, it remains one of the most important causes of infant morbidity and mortality (1, 2). Its worldwide incidence is estimated at 20/1,000 births (3) but varies strongly from region to region, with resource-limited countries having a more significant incidence. Even in countries with the highest levels of healthcare, 1–5/1,000 full-term infants develop hypoxic-ischemic encephalopathy (HIE), an encephalopathy indisputably attributed to hypoxic brain injury (4).

HIE is associated with a high death rate and long-term morbidity (5). It has been proven and widely accepted that the use of therapeutic hypothermia (TH) performed within the first 6 h of life for a duration of 72 h, is associated with a significant reduction in death and disability at 18 months (6–11). This is currently the only treatment modality validated in moderate and severe HIE cases. TH is not currently standard treatment in mild encephalopathy; and a percentage of these neonates may, albeit with mild symptoms at birth, develop disabilities or cognitive abnormalities. TH may indeed be beneficial in these mild cases.

The discovery and validation of neurobiomarkers could be useful and add value as a screening tool, to stratify the different severity grades of HIE, and objectively assist in the criteria for TH. Furthermore, having diagnostic and prognostic biomarkers of severity, may lead to a more proactive approach in the use of adjunctive therapies other than hypothermia, such as melatonin or erythropoietin (12, 13). Ideal screening biomarkers should be easy to collect, have a fast laboratory turnaround time, be reliable and inexpensive. Current research on micro-RNAs, certain proteins and single metabolites has not identified an ideal biomarker fulfilling these criteria.

Metabolomics; defined as the study of low molecular weight metabolites present in the body, seems to be a promising novel approach in the search of neurobiomarkers. Metabolites analyzed may be peptides, lipids, organic acids, vitamins, minerals, drugs, amino acids, nucleic acids, carbohydrates, fatty acids, hormones, drugs, and any other chemicals with a molecular weight <2,000 Da. Metabolomic identification can be achieved using two techniques: a targeted and a non-targeted approach. The first approach consists of investigating pre-known and expected metabolites. The non-targeted approach conversely investigates all metabolites detectable in a sample, aiming to capture as much information as possible, providing a functional “fingerprint” of the pathological state being investigated. This metabolic fingerprint may then be validated in different experimental groups as a screening biomarker (14).

The use of metabolomics in HIE is 2-fold: firstly, adding to the understanding of biological pathways involved in HIE, and secondly creating a panel of metabolites that may act as this “fingerprint” characterizing the levels of severity in various HIE cases.

Several metabolomic studies have been performed over the last years studying various biological fluids. No specific neurobiomarkers to date have been validated for clinical use. The aim of this review is to analyze the most promising studies in the field of neurobiomarkers arising from metabolomics, in neonatal HIE, in order to improve diagnosis and prognostication in this group of infants.

## Methods

### Search Strategy and Selection Criteria

The review was conducted through a database search of PubMed and the Cochrane Library from January 1976 to February 2021.

The first search included the terms “metabolomics” AND “neonatal asphyxia” OR “perinatal asphyxia” OR “HIE” OR “hypoxic ischemic encephalopathy” OR “hypoxic ischemic encephalopathy.” The second search included the terms “neurobiomarkers” AND “neonatal asphyxia” OR “perinatal asphyxia” OR “hypoxic ischemic encephalopathy” OR “hypoxic ischemic encephalopathy.” Only English-language, peer-reviewed studies were included. Case reports and conference abstracts were excluded. We considered the studies eligible if they included term/late preterm infants (≥35 weeks gestational age) diagnosed with perinatal asphyxia.

### Data Extraction

**Figure 1:**
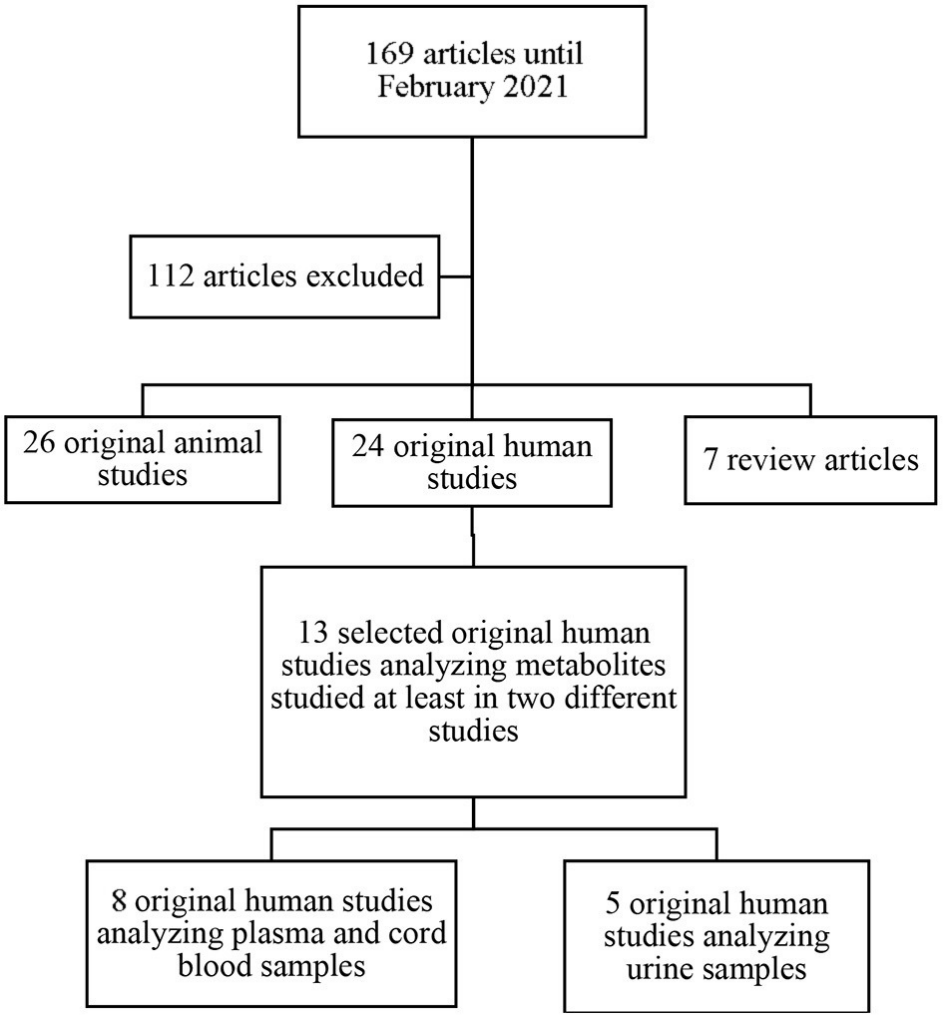
Search strategy used and selection of studies.

The search identified 169 potentially relevant papers fitting the search criteria. After title and abstract screening, 57 full-text studies were eligible for inclusion (Figure 1). Of the 57 studies included; 7 were review articles, 24 were original human studies, and 26 were original animal studies. We excluded the 7 review papers. After exclusion of articles not meeting inclusion criteria, we included 13 original human studies. The methods of metabolomic analysis are presented in Table 1.

**Table 1:** Studies assessing metabolites as biomarkers to predict neurological outcome in asphyxic neonates with/without HIE.

**Studied population**	**Compared groups**		**Timeline of the analysis**	**Biological fluid**	**Authors**
Neonates with HIE (managed with hypothermia)	HIE	Matched controls	48 h after topiramate (TPM) or placebo administration	Plasma	(22)
	Metabolomic changes over time during HT		Birth, 24, 48, and 72 h after the administration of TPM or placebo	Cord blood + plasma	(22)
	Normal MRI day 4 to 8	Pathological MRI at day 4 to 8	Birth, 24, 48, and 72 h after the administration of TPM or placebo	Cord blood + plasma	(46)
Asphyxic Neonates (No HIE diagnosis)	Asphyxia without HIE	Matched controls	Birth	Cord blood serum	(29, 31, 54, 55)
	HIE without asphyxia	Matched controls	Birth	Cord blood serum	(29, 31, 54, 55)
	Asphyxia with HIE	Matched controls	Birth	Cord blood dried spot	(56)
	Asphyxia without HIE	HIE without asphyxia	Birth	Cord blood dried spot	(56)
	Asphyxia without HIE	HIE without asphyxia	Birth	Cord blood serum	(31, 54)
	HIE grade 3	Matched controls	Birth	Cord blood serum	(29)
	HIE grade 3	Asphyxia without HIE	Birth	Cord blood serum	(29)
	HIE severity grade		Birth	Cord blood serum	(29, 54)
	Norma/several outcome		Birth	Cord blood serum	(47)
Neonates with HIE (managed with hypothermia)	T1 (48 h)	Baseline = sample at birth = T0	5 samples: birth (T0), after 48 h HT (T1), after 72 h HT (T2), after 1 w (T3), after 1 m (T4)	Urine	(50)
	T2 (72 h)	Baseline = sample at birth = T0			
	T3 (1 week)	Baseline = sample at birth = T0			
	T4 (1 month)	Baseline = sample at birth = T0			
	HIE at birth	Healthy controls at birth	4 samples: birth, day 2, day 3, day 30	Urine	(49)
	HIE at day 3	HIE at birth			
	HIE at day 30	HIE at birth			
Asphyxic Neonates (No HIE diagnosis)	Asphyxia and HIE or perinatal death	Asphyxiated (without HIE) and controls	2 samples: within 12 h and between 12 and 48 h	Urine	(69)
	Asphyxia without HIE	Asphyxiated and HIE or perinatal death and controls			
	Asphyxia with HIE	Matched controls	2 samples: day 1, day 2	Urine	(48)
	HIE with/without HT	Matched controls	3 samples: day 1, day 3, day 9	Urine	(53)

Animal studies were taken into consideration in order to support or qualify the findings from human studies.

It is clear from the literature and the reviews that an extensive number of biomarkers and metabolites have been studied. There remains no consensus however on the specific use of these neurobiomarkers in clinical practice.

It is important to note that the review articles already existing, included animal studies and these results may not be significant or translatable in humans (15–17). One review presented a range of biomarkers including RNA and cytokines and did not focus on metabolites specifically (18). The most recent review by Bardanzellu and Fanos (19) in 2020 reviewed the use of metabolomics in pediatrics, however did not focus specifically on infants with asphyxia and encephalopathy due to hypoxic-ischemia. The two reviews “most similar” to our review, were published in 2010 and 2015, respectively (3, 20), and in an ever changing and dynamic research setting; we believe that there have been advances in techniques and interpretation worth reviewing.

In our review, we included human studies only with specific metabolites analyzed in at least two different studies. This method was chosen not only to narrow the spectrum of metabolites presented, but also to exclude possible non-significant metabolites found by chance. By doing so we feel this “fine-tuned” the proposed metabolites suggested for the screening process in asphyxiated infants. This proposed screening “tool” could be used/tested within the first hours of life in infants with asphyxia to ascertain which infants would develop encephalopathy and which would develop long-term poor outcome.

To our knowledge, this is the first review to present to clinicians and researchers, the metabolites across at least two studies show the greatest promise in differentiating and predicting the severity level and prognosis of infants born with asphyxia and hypoxic ischemic encephalopathy.

## Results

The characteristics of the studies included are reported in Table 1, with an outline of the various metabolites considered as potential neurobiomarkers in HIE.

Amongst the 13 articles reviewed, two analyzed biological data of patients included in the HYPOTOP trial cohort—a randomized, controlled, multicenter, double-blinded clinical trial aiming to assess the efficacy of topiramate vs. placebo in patients with HIE undergoing TH (21). Sanchez-Illana et al. (22) performed a study on this cohort analyzing metabolites involved in neurological pathways and they did not find any effect of the administration of topiramate on metabolite levels. It is for this reason we decided to include patients involved in the HYPOTOP trial in our review.

The most common metabolites considered as potential biomarkers, include energy related metabolites (lactate, pyruvate, glycerol, N-acetylglucosamine, glucose, galactose, and lactose), amines, amino acids and amino acid metabolism intermediates (acylcarnitines, ketone bodies, metabolites of the Krebs cycle, steroid hormone biosynthesis metabolites), and other metabolites measured in urine (aconitate, formate, and kynurenic acid). The various metabolites/potential neurobiomarkers in HIE are described in detail below.

### Energy Related Key Metabolites

#### Lactate

Lactic acid is the final breakdown product of pyruvate during anaerobic metabolism. Lactate levels rise during hypoxia due to the shift from the aerobic metabolism to anaerobic metabolism. Lactate has been used for decades as a marker of poor tissue perfusion in newborns suffering from asphyxia and is highlighted in many studies performed both in humans (22–24) and animals (25–28).

#### Blood Levels

Blood lactate levels are significantly higher amongst newborns suffering from asphyxia (with or without signs of HIE) compared with controls (22, 29–31). It has been suggested that a lactate concentration of >8 mmol/L is a good indicator of intrapartum asphyxia when associated with a base deficit of >-12 mmol/L (32). Lactate levels alone however do not distinguish between asphyxiated neonates who develop HIE from those who recover (29, 31). Solberg et al. described in a population of newborn piglets, that the plasma lactate levels do not always correlate with hypoxia duration and significance (33).

Da Silva et al. established that a blood lactate level >9 mmol/L at 30 min of life was associated with a moderate to severe clinical neurologic evolution (according to Finer's classification) with a sensitivity of 84% and a specificity of 67% (34). A level <5 mmol/L was however associated with no risk of developing clinical signs of encephalopathy (35). In contrast, a study performed by Murray et al. showed that the initial rise of blood lactate observed in neonates with asphyxia could not be used to predict the gravity of the subsequent encephalopathy (36).

The persistence of a lactic acidosis has however been associated with severe encephalopathic patterns on EEG monitoring. After an initial increase, the lactate level should decrease gradually and normalize (22, 37). Shah et al. demonstrated that lactate levels normalize slower in asphyxiated newborns with moderate to severe encephalopathy in comparison to those presenting with mild encephalopathy (38). Even in asphyxiated lambs prolonged lactic acidosis was associated with important brain injury (39, 40). The clearance delay could be related to hepatic and renal dysfunction (41–44), or additional production of lactate due to convulsive activity (36). In terms of prognosis, Frey et al. showed that asphyxiated neonates who died during the first week of life had significantly higher lactate levels than those presenting with more mild neurological deficits (45). Pineiro-Ramos et al. however, could not demonstrate a relation between the cord blood lactate levels of neonates presenting with HIE and the severity of pathological cerebral magnetic resonance imaging (MRI) performed between day 4 and 8 of life (46). Ahearne et al. also showed that cord blood lactate levels in asphyxiated neonates did not predict outcome at 3 years of age (47).

#### Urinary Levels

Some studies have highlighted a higher urinary level of lactate in asphyxiated neonates (with and without HIE) in comparison to controls (48, 49). Urinary lactate levels generally decrease progressively with time (up to 1 month after birth) in neonates suffering from HIE (49, 50).

#### Cerebrospinal Fluid Levels

Magnetic resonance spectroscopy (MRS) studies suggest that high blood lactate levels after neonatal asphyxia, arise from altered brain metabolism. In fact, in neonates with HIE who died or developed severe neurological disability, high levels of lactate were shown both on cerebral proton magnetic resonance spectroscopy (51) and on the cerebrospinal fluid measurements (23). Van Cappellen et al. detected the presence of significantly higher levels of lactate in the cerebrospinal fluid of severely asphyxiated fetal lambs in comparison with a control group (52).

In summary, the increase and especially the persistence of high blood lactate levels, seem to reflect the degree of fetal hypoxia, but do not give clear predictive information on the risk of developing an adverse neurological outcome (18). As stated by Bennet et al. (20), it remains difficult to differentiate patients on the moderate to severe encephalopathy spectrum.

### Pyruvate

#### Blood Levels

Reinke et al. observed that pyruvate cord blood levels were higher in asphyxiated neonates, with or without HIE compared to controls (29). Pyruvate levels did not however predict the severity of the grade of asphyxia, as the authors could not find a significant difference between neonates presenting with mild, moderate and severe HIE (Sarnat 3) compared to the control group. Even in the HYPOTOP trial, the analysis of pyruvate cord blood levels among neonates presenting with HIE and treated with TH, did not identify and differentiate those with pathological brain MRI findings (46). There were higher pyruvate levels 48 h after the administration of topiramate in neonates presenting with HIE and treated with TH, in comparison to the control group. The level of pyruvate seems to decrease gradually during TH (22) and in an animal study (27) pyruvate blood levels were indeed higher 2 h post the asphyxic episode, decreasing progressively 4 h after the event.

In the HYPOTOP study, the pyruvate levels collected at 24 and 48 h in patients with HIE treated with TH, did not predict the extent of pathology on cerebral MRI performed between day 4 and 8 of life. In samples collected 72 h after the administration of topiramate, the pyruvate level was however significantly higher in the group with a pathologic MRI (46).

#### Urinary Levels

Locci et al. found the urine pyruvate levels at birth lower in neonates presenting with HIE, in comparison to a control group (49). On the contrary, on the third and 30th day of life, the neonates with HIE treated with TH had higher urinary pyruvate level compared with levels measured at birth. Sarafidis et al. also observed higher urinary pyruvate levels in newborns with HIE (treated and non-treated with TH), compared to controls at 3 days of life (53).

### Glycerol

#### Cord Blood Levels

Two studies observed glycerol cord blood levels higher in patients with asphyxia (with or without HIE) than controls (29, 54). Reinke et al. (29) found that glycerol levels were significantly higher in neonates with asphyxia compared to controls, but only in the severe HIE (grade 3) subset. Similar findings were observed in an animal study performed on macaques (28). On the contrary, Denihan et al. (54) found no difference in the glycerol levels despite the severity of HIE.

### N-Acetylglucosamine

Studies measuring urinary N-acetylglucosamine are discordant. Noto et al. studied neonates with HIE (treated with TH) and found lower levels of urinary N-acetylglucosamine measured at 48 and 72 h, in comparison to levels measured at birth (50). The opposite was demonstrated by Locci et al., where a significant increase was found on day 3 and 30 of life in comparison to birth levels (49). In neonates with HIE, the levels measured at birth were lower than in the healthy control group.

### Glucose

The studies performed by Sachse et al. and Longini et al. highlight significant glycosuria in asphyxiated patients (with or without HIE) in comparison with control groups (levels were carried out daily for the first 2 days of life) (27, 48). Locci et al. did not confirm this finding at birth, however urinary glucose was higher on the third day of life in patients with HIE (49). It is speculated that the asphyxic event leads to glycosuria, and although may not be evident in the first day of life, may appear in the following days.

### Galactose and Lactose

These two metabolites can be analyzed in the urine. Noto et al. have shown in neonates with HIE treated with TH, that levels were lower when measured after 72 h, 1 week, and 1 month of life for galactose; and after 48 h, 1 week, and 1 month of life for the lactose; in comparison to levels measured at birth (50). Locci et al. found the opposite finding (49) and no differences at birth between neonates with HIE and the control group.

In summary, concerning these energy related metabolites; pyruvate, glycerol, N-acetylglucosamine, glucose, galactose, and lactose, several studies have demonstrated different levels between asphyxiated neonates and healthy controls, and none of the levels (collected in blood or urine and carried out at birth or later) showed significance in relation to the severity of asphyxia.

Regarding prognosis in HIE, only blood pyruvate levels (72 h after administration of the topiramate in the HYPOTOP study) allowed some prediction of cerebral MRI outcome, performed between day 4 and 8 of life.

### Amines, Amino Acids, and Amino Acid Metabolism Intermediates

#### Blood Levels

Amino acid levels analyzed from cord blood have been observed to be significantly higher in patients suffering from asphyxia in comparison to control groups. Among these amino acids according to various studies, alanine, creatinine, isoleucine, leucine, methionine, phenylalaline, taurine, and valine are the most significant (cf. Table 2) (29, 31, 54–56). The same finding has been observed in several animal studies (25, 27, 28, 33). Of note, only methionine levels showed the same trend systematically across the different studies and only alanine was found to be significantly higher in patients with severe HIE (grade 3) in comparison to asphyxiated patients without HIE and the control groups (29). Some amino acid levels were also significantly different amongst asphyxiated neonates who went on to develop HIE vs. those who did not (29, 56). The levels of alanine, phenylalanine, valine and leucine were significantly higher in the HIE group studied by El-Farghali et al. (56).

**Table 2:** Variation of cord blood metabolites.

**Biomarker**	**References**	**Levels associated with Asphyxia**	**Levels associated with HIE**
Lactic acid	(22, 31)	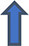	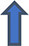
Creatine	(29, 54)	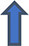	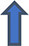
Creatinine	(29, 54)	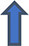	
Carnitine	(29, 31, 54)	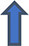	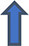
Alanine	(29, 55, 56)	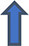	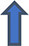
Isoleucine	(29, 55)	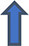	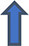
Isoleucine	(29, 55)	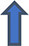	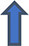
Leucine	(29, 54–56)	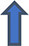	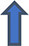
Methionine	(29, 31, 55, 56)	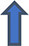	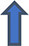
Phenylalanine	(29, 31, 54–56)	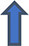	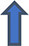
Valine	(29, 55, 56)	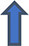	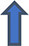
L-acetylcarnitine [C2]	(54–56)	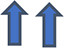	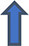
Hydroxybutyrylcarnitine	(55, 56)	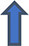	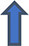
Butyrylcarnitine [C4]	(55, 56)	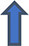	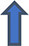
Tetradecanoylcarnitine [C14]	(55, 56)	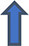	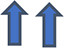
L-Palmitoylcarnitine [C16]	(55, 56)	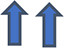	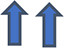
Hexadecenoylcarnitine [C16:1]	(55, 56)	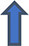	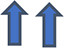
Stearoylcarnitine [C18]	(55, 56)	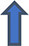	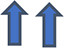
Oleoylcarnitine [C18:1]	(55, 56)	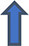	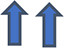
Pyruvate	(22, 29, 46)	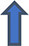	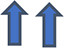
Hydroxybutyrate	(22, 29)	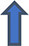	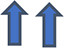

No difference was observed with the levels of creatinine, isoleucine and the methionine.

#### Urinary Levels

In the study of Longini et al. (48), urinary aspartate and threonine levels measured within the first 2 days of life were significantly higher in asphyxiated neonates (with and without HIE) in comparison with a control group. In contrast, Sarafidis et al. (53) found that patients with HIE (treated and not treated with TH), had lower urinary levels of these two urinary amino acids on the first day of life compared to control group. On the contrary, no difference was highlighted between those two groups when the levels were measured after 3 and 9 days of life (53).

Longini et al. found the urinary levels of betaine, creatinine and dimethylglycine significantly lower in asphyxiated children (with or without HIE) in comparison to a control group during the two first days of life (48). However, differences have been highlighted in other publications between these 3 metabolites. Noto et al. showed an increase of urinary creatinine in neonates presenting with HIE (treated with TH) at the end of treatment and at 1 week of life (50) and Locci et al. found increases only in betaine at birth in patients presenting with HIE in comparison to the control group (49). In this study, the evolution of betaine in neonates with HIE treated with TH is characterized by a decrease at day 3 of life followed by an increase at day 30 of life. Creatinine levels present an opposite evolution with an increase at day 3 of life and a decrease at day 30 of life (in comparison to the level measured at birth). The level of dimethylglycine however, remains elevated at day 3 and day 30 of life (in comparison to the level measured at birth).

Longini et al. (48) also analyzed dimethylamine in the urine during the first 2 days of life; and found the level to be significantly lower in asphyxiated neonates (with or without HIE) in comparison to a control group. This tendency was confirmed by Locci et al. (49), demonstrating lower dimethylamine levels at birth in patients presenting a HIE in comparison to the control group. Within the same group of HIE infants, there was a significant increase of dimethylamine levels at the end of TH and at 30 days of life, in comparison to the level measured at birth.

Neonates with asphyxia and HIE have higher levels of urinary taurine at birth compared to controls. Locci et al. highlight a constant decline in levels during the 72 h of TH (49). Noto et al. observed the same tendency (50) with a decrease measured at 1 week and 1 month after birth.

In summary, although amines, amino acids and amino acid metabolism intermediate levels (carried out at birth and/or thereafter and measured in the blood and/or in urine), may show differences in levels between asphyxiated neonates and healthy controls across several studies, they do not predict severity or outcome in HIE.

### Acylcarnitines

The acylcarnitines reviewed and mentioned are represented by their chemical nomenclature which is shown between brackets (e.g., Oleoylcarnitine is [C18:1]).

#### Blood Levels

Studies performed in animals have highlighted higher levels of acylcarnitines after hypoxia (33, 57). Similarly, the cord blood levels of several acylcarnitines were significantly higher in asphyxiated neonates in comparison to control groups (cf. Table 2).

In particular, L-acetylcarnitine [C2] and Butyrylcarnitine [C4] blood levels have been shown to be higher in asphyxiated neonates (with or without HIE) in comparison to control groups (31, 55, 56). These levels did not however identify those at risk of developing HIE (54, 56) or predict the grade of severity of HIE in which the infant would be (54).

The level of Hydroxybutyrylcarnitine (Malonylcarnitine) [C3-DC (C4-OH)], Tetradecanoylcarnitine [C14], L-Palmitoylcarnitine [C16], Hexadecenoylcarnitine [C16:1], Stearoylcarnitine [C18], and Oleoylcarnitine [C18:1] were not only significantly higher in asphyxiated patients in comparison with control groups (31, 55, 56), but were also significantly higher in the group of asphyxiated patients who developed HIE in comparison to those who did not develop HIE (56).

### Ketone Bodies

#### Blood Levels

Levels of acetone and β-hydroxybutyrate analyzed on cord blood (29, 54) have been found to be higher in asphyxiated patients without HIE in comparison to the control group. Oddly however levels were not significantly different when comparing patients with HIE to the control group. Only in the study of Reinke et al. (29) were the levels of acetone and β-hydroxybutyrate measured on cord blood higher in neonates with severe HIE (grade 3) compared to asphyxiated neonates without HIE.

Furthermore, the HYPOTOP study revealed a significant decrease in the level of β-hydroxybutyrate 48 h after the injection of topiramate in the group of patients presenting with HIE and treated with TH in comparison to the control group (22). A progressive decrease was observed during TH.

#### Urinary Levels

Urinary acetate levels, in the context of neonatal hypoxia, have been performed in the following two studies; Longini et al. and Locci et al. Longini et al. highlighted lower levels of ketone bodies in asphyxiated neonates (with or without HIE) in an analysis performed during the two first days of life, in comparison to a healthy control group (48). The same was found by Locci et al. in a cohort of neonates at birth presenting with HIE (49).

In summary, even though the ketone body levels have differed in several studies between asphyxiated neonates and healthy controls, none of the considered levels (carried out at birth and measured on cord blood or later measured in urine) enabled prediction of the severity of HIE.

### Metabolites of the Krebs Cycle

#### Blood Levels

In the studies of Reinke et al. (29) and O'Boyle et al. (31), the succinate levels measured on cord blood were higher in asphyxiated neonates (with or without HIE) than in the control group. This finding has also been observed in several studies performed in animals (27, 28, 33, 58). Furthermore, a significant difference was found between asphyxiated neonates presenting with HIE (independent of grade) and those without HIE.

Denihan et al. however did not reach the same conclusion. Using untargeted metabolomics, they analyzed a metabolite with features similar to succinate (but also Methylmalonate or Threonolactone). This analysis revealed an increase of the metabolite among the group of asphyxiated neonates who did not develop HIE, but not in the group presenting with HIE (when compared to the control group) (54). Furthermore, even though these levels were statistically significant in the various severity groups of HIE, this difference did not remain significant following correction for confounding factors.

In the study of Sanchez-Illana et al. (HYPOTOP study) (22), there was no difference in the succinate levels measured 48 h after administration of topiramate in neonates presenting with HIE (treated with TH), in comparison to the control group. The succinate levels in the HIE group remained stable during throughout TH.

#### Urine Levels

Citrate and succinate levels have been studied in urine too. These two metabolites were found in lower levels in asphyxiated patients (with or without HIE) in comparison to the control group (48, 49).

In summary, even though differences in the levels of metabolites of the Krebs cycle were found in several studies, none of the observed levels predicted the severity of HIE.

### Steroid Hormone Biosynthesis Metabolites

Among all the steroid hormone biosynthesis metabolites studied by Pineiro-Ramos et al. (46), only dehydroepiandrosterone had been analyzed in a second study by Denihan et al. (54). It is important however to note that the latter study was performed using untargeted metabolomics and the authors analyzed a metabolite with features matching dehydroepiandrosterone sulfate but also epitestosterone sulfate or testosterone sulfate.

Pineiro-Ramos et al. highlighted an increase in the levels of dehydroepiandrosterone, in neonates with HIE (treated with TH), collected on cord blood and at 24, 48, and 72 h after the administration of topiramate (HYPOTOP study). These neonates had pathological MRIs between day 4 and 8 of life. Denihan et al. showed a significant decrease of dehydroepiandrosterone levels in asphyxiated neonates without HIE compared to a control group of neonates without asphyxia.

In summary, the limited number of studies analyzing dehydroepiandrosterone as a neurobiomarker, and the findings arising from these studies, do not validate its efficiency in differentiating neonates with HIE from control neonates. Furthermore, to our knowledge, no studies in animals have investigated this biomarker.

### Purines and Pyrimidine Derivatives

Denihan et al. (54) and O'Boyle et al. (31) found a significant increase in uridine levels measured on cord blood of asphyxiated neonates with and without HIE, compared to a control group of neonates without asphyxia. Both studies however did not find any difference between asphyxiated patients with HIE and those without HIE, making the use of this metabolite less useful.

### Other Metabolites Measured in Urine

#### Aconitate and Formate

Longini et al. reported lower levels of aconitate and formate, two urinary metabolites, during the first 2 days of life, in asphyxiated neonates with or without HIE, in comparison to a control group. There was no difference however at birth between patients with HIE and the control group (48, 49). The levels were higher after 30 days of life (in comparison to the level measured at birth) in patients presenting with HIE and treated with TH (49). Even at 3 days of life, the aconitate levels were higher than birth levels in the HIE group (49). This was not seen in the formate levels.

#### Kynurenic Acid

Sarafidis et al. showed a lower level of kynurenic acid on the first day of life, in neonates presenting with HIE (with or without treatment with TH), in comparison to a control group (53). This difference disappeared after the third day of life. Noto et al. highlighted a decrease in kynurenic acid levels at 48 and 72 h, and 1 month of life in a population of neonates suffering from HIE and treated with TH, in comparison with their levels measured at birth (50).

The limited number of studies analyzing this metabolite leaves unanswered questions regarding its utility.

In summary, neither aconitate and formate, nor kynurenic acid levels seem to accurately predict the degree of severity of asphyxia in infants with HIE.

## Discussion

The pathophysiological mechanisms behind HIE are complex, varied and at times not well-understood. Many factors such as the etiology, the duration and the severity of hypoxia, and the regional variations of cerebral blood flow can influence the nature and the extent of the brain injury as well as the outcome of the affected neonate. Animal models have allowed us to understand that the initial damage caused by the switch to anaerobic metabolism (primary energy failure) produces an immediate cell death. The restoration of cerebral blood flow after the ischemic insult leads to an alteration of metabolism (the secondary energy failure) provoking an inflammatory cascade. This inflammatory phase is characterized by the production of reactive oxygen species (free-radicals), the formation of proteases and caspases and leads to excitotoxicity, apoptosis and neuronal cell death. This second phase occurs after a latent period of 6 to 48 h (59).

The pathophysiology of HIE is strongly dependent on the perturbation of the different mechanisms of compensation, which usually withstand the “physiological” hypoxia found during labor and delivery. The neonatal brain is highly dependent on oxygen and has a low concentration of antioxidants, but a high concentration of unsaturated fatty acids. Together, these characteristics make the neonatal brain vulnerable to permanent damage, if critical levels of energy deprivation are reached (60), leading to a wide spectrum of neurological deficits.

TH is the only evidenced based treatment available to date to reduce the incidence of neurological impairment due to asphyxia at birth. Hypothermia has proven to be effective especially in moderate to severe cases of asphyxia. Nevertheless, it is not always easy after the ischemic event, to determine and accurately predict in which severity group the neonates may be and especially in the infants with mild asphyxia, who may be at risk of developing neurological impairment. With this in mind, the aim of investigating and validating neurobiomarkers to identify neonates at risk is highly suitable.

Metabolomics is promising is the field of biomarker identification. It consists of the quantitative analysis of many low molecular mass metabolites, involving both substrates and products of particular metabolic pathways, which may identify key metabolite levels (raised or decreased) due to the intricate interaction between the pathophysiological state, gene expression, and environment.

A study performed in adults highlighted that hypoxia has a dose-dependent effect on the metabolic composition in urine (61). It is intuitive that in cases of severe hypoxia there will be a modification of the metabolic milieu. In cases of severe neonatal hypoxia, the use of neurobiomarkers may seem less attractive, as TH is clearly indicated in these severe cases. It is however in the cases with mild hypoxia, where the current treatment guidelines do not promote/advocate for TH, that studies are now reporting in the region of 20% of cases having adverse neurological outcomes at a later stage (62). Brain imaging of these infants with mild encephalopathy may show detectable abnormalities on MRI (63, 64). It is therefore becoming more evident that the grade of neonatal encephalopathy during the first hours of life does not always adequately identify the children prone to develop cerebral injury.

Different biofluids can be used for the identification of neurobiomarkers. Blood measurements allow fast and often real-time information, but blood collection in neonates may be difficult to perform and remains relatively invasive given their low total blood volume. Cord blood is easy to collect and is available in large volumes allowing multiple analyses, however only analyzes the situation at the moment of delivery. The studies of cord blood biomarkers combined with subsequent blood plasma analysis prove to provide a more interesting and inclusive approach.

The collection of urine in theory is easy and non-invasive, but may depend on the renal function of the neonate and in particular, infants with perinatal asphyxia are often oliguric or anuric and it may therefore take several hours to collect sufficient quantities of urine. This could represent a problem, as the goal of the neurobiomarker identification is (within the first 6 h of life) to ascertain whether or not the infant will benefit from TH and to expedite the treatment.

The evidence of analyzing saliva as a biofluid in neonates is sparse in the literature, and although it has the advantage of being easy to collect and non-invasive, it may be limited in quantity at times.

The analysis of cerebrospinal fluid may be crucial for the diagnosis and management of certain neurological diseases. Lumbar punctures are however invasive and not always easy to perform in neonates. The quantity of fluid obtained may also be limited.

There are indeed numerous metabolites that have been studied, reviewed and reported as proposed neurobiomarkers. Skappak et al. highlighted that whilst they found 13 urinary metabolites associated with hypoxia in piglets (in comparison to the control group), if these were studied independently, the metabolites were not found to be significant. When the 13 metabolites were combined however, they had an accuracy of 90% (25). This reiterates that often the most effective way to interpret metabolomics, is through the identification of a particular “fingerprint,” composed of various metabolites expressed together, in a clinical setting such as asphyxia.

Many studies have shown that hypoxic neonates exhibit increased metabolites derived from lipid peroxidation (65). These measurements however, have appeared to be ineffective in identifying those who will develop HIE. A common finding across studies was that neonates with severe HIE are unable to produce neuroprotective ketones (22, 29). The metabolites proving to be most promising in identifying neonates who will develop HIE, are the group of acylcarnitines: Hydroxybutyrylcarnitine (Malonylcarnitine) [C3-DC (C4-OH)], Tetradecanoylcarnitine [C14], L-Palmitoylcarnitine [C16], Hexadecenoylcarnitine [C16:1], Stearoylcarnitine [C18], and Oleoylcarnitine [C18:1]. In the studies of Walsh and El-Farghali the metabolites mentioned above were not only significantly higher in the cord blood of asphyxiated neonates compared to normal controls, but were significantly higher in the group presenting with HIE compared to the group of asphyxiated patients who did not present with HIE (55, 56). From a biological point of view, this could be explained by the reduced level of β-oxidation of long chain acyl-CoA due to the occurrence of hypoxia, which seems to induce the accumulation of its precursor: acylcarnitines (16). This early screening measurement thus seems promising, as it may allow early identification of neonates who would benefit from TH.

A promising metabolomic “index,” created to identify the neonates at risk of developing HIE, was proposed by Reinke et al. In their study, several metabolites associated with HIE, were measured on cord blood, and stepwise multiple linear regression showed that 3-hydroxybutyrate, glycerol, O-phosphocholine, and succinate were most predictive of HIE severity. The “index” used was the following: (succinate x glycerol) / (β-hydroxybutyrate × O-phosphocholine) (29).

This index was subsequently validated in a study by Ahearne et al. (47). They analyzed the index in relation to the outcome at 3 years of age in a population of asphyxiated neonates (including those with and without HIE). The 3 year outcome used was the Bayley Scales of Infant and Toddler Development, ed. III (BSID-III). The “severe” outcome group was defined as those infants with the outcomes of death, dyskinetic, or spastic quadriplegic cerebral palsy or ≤70 in all BSID-III subscales. The study revealed that a metabolite index of <0.13 was associated with normal outcome (sensitivity 65% and specificity of 91%), whereas an index of >2.4 was associated with a “severe outcome” (sensitivity of 80% and specificity of 100%). This index method needs to be validated on urine as due to the relative ease of collection, an increased sampling opportunity is available.

The use of this metabolite index is the first attempt combining several metabolites in order to analyze the blood metabolome in asphyxiated newborns. The study of these complex biomarker relationships may assist for diagnostic and prognostic purposes. This approach seems promising and validation in larger groups of asphyxiated neonates is needed to provide further evidence of its reliability, and its implication in predicting outcome in this vulnerable group of neonates.

Ahearne et al. (47) suggested that the biological plausibility of this metabolomic index increases the probability of its predictive potential. Indeed, hypoxia-ischemia leads to changes in cellular energy pathway molecules, such as glycerol and succinate, through mitochondrial dysfunction and subsequent disruption of the tricarboxylic acid cycle (33). O-phosphocholine is an anabolic and catabolic metabolite of cell membrane metabolism, and together with glycerol may indicate cell membrane degradation (66). The modification of the levels of O-phosphocholine has been previously described in animal models of hypoxia-ischemia (67). The ability of the brain to upregulate ketone metabolism to provide an alternative energy source is an important means of brain adaptation in early life (68). This is the reason why the contribution of hydroxybutyrate to the model may indicate defective ketone body production, complete consumption of the ketone body supply, or inadequate prior adaptation to ketone body metabolism, causing the insurmountable secondary energy failure leading to long-term damage (47).

Recently, O'Boyle et al. also tried to combine several metabolites in order to produce a snapshot of the expected blood metabolome in asphyxiated newborns at birth (31). Unfortunately, umbilical cord blood metabolites alone were not sufficiently predictive of the development of HIE in asphyxiated neonates. For this reason, they added clinical data to these measurements. In summary, they showed that the clinical condition at birth (Apgar scores at 1 and 5 min, details of intensive resuscitation required at birth, and the need for assisted ventilation at 10 min of age), combined with both lactic acid and alanine measurements could be used to give the optimum model for distinguishing between neonates with perinatal asphyxia alone and those who would go on to develop HIE. Alanine plays a key role in gluconeogenesis in mammals and is also the result of the transamination of pyruvate (deriving from lactate). It is for this reason the increase is significant as it provides evidence of the transitional state to anaerobic metabolism. It is therefore not surprising that a rise in circulating alanine is seen in asphyxiated neonates. The significance of the increase of lactate during hypoxia has been explained previously.

### Limitations and Strengths

In order to critically appraise this review, one needs to acknowledge the limitations and the strengths of the review. One of the main limitations is that the effects of TH itself on the metabolic pattern were not explored and discussed here. The decrease of body temperature is responsible for the slowing of the metabolism and can alter enzymatic activity and therefore the production of different metabolites. This may influence and lead to complex effects on multiple regulatory processes.

The feeding and infusion regimens of the neonates in the studies reviewed was also not discussed, both of which could affect the metabolic pattern. It is well-described and practiced that neonates undergoing TH are kept nil per os or with minimal enteral feeding and this effect on the metabolite results may be negligible. This may not be the case for parenteral nutrition.

It is true that whilst most of the reviewed articles measured the metabolites on cord blood at birth, there were some measurements within 72 h (and later). No specific mention was made of the feeding regimes. Receiving parenteral nutrition may have influenced these metabolite levels although most parenteral nutrition solutions used in the neonatal intensive care units have standardized protein, fat and carbohydrate components and are standardized for each day of life.

In the design of future studies, it would be important to take this factor into account by reviewing the volume/kg of parenteral nutrition received and or other fluids/feeds received, when analyzing the data.

We are specifically interested in the metabolite levels within the first 6 h of life, as it is within this crucial window that the clinicians need to decide which infants would benefit from therapeutic hypothermia. At this early stage, parenteral nutrition has not generally been commenced. If one measured the metabolites in the infants with asphyxia at a later stage (for example day 2, 3 onwards), this would need to carefully considered.

Another important point which may be viewed as a limitation, is the inclusion of studies that analyzed data from the HYPOTOP trial cohort. The reasons for including these papers have been described. Finally, given the extensive number of metabolites studied in the current literature, the decision to review and include only the articles with metabolites analyzed in at least two different studies was decided arbitrarily, which may lead to bias or important missed metabolites not reviewed.

The review also presents strengths as the current literature has a significant number of metabolites studied in the context of HIE. To our knowledge, this review is the first to present to clinicians and researchers in a concrete manner, the metabolites that, according to several studies, seem to have the greatest ability to differentiate the degree of severity of asphyxia in these patients.

## Conclusion

There is currently early preliminary knowledge of the neurobiomarkers that may assist physicians in improving the selection criteria for therapeutic hypothermia after perinatal asphyxia. It appears these early validation results, using a panel of neurobiomarkers acting together as a “fingerprint,” may add information to help categorize infants into HIE severity groups, improve and guide management with TH, as well as aid as a prognostic tool.

Metabolomics, a novel methodology in the development of neurobiomarkers, ideally measured within the first 6 h of life, may be able to identify neonates who will develop, without further intervention, encephalopathy. Despite a bedside method to measure these metabolites not being currently available, considering the increasing and keen interest of metabolomics it seems realistic and possible for future “bench to the bedside” analysis advances. Not only will this add vital additional information for neonates considered as having “mild asphyxia” (where in most centers worldwide TH is not offered), but will lead to more prompt treatment in all neonates at risk. The dynamic rate of the biomarker evolution also will add additional information for prognostic value.

As mentioned, in order to produce and provide a safe and effective screening tool, the collection of samples needs to be easy, the analysis reliable and the costs kept to a minimum. As shown in this review, no single metabolite seems to fulfill these criteria. In the future, combinations of metabolites used as a biomarker “fingerprint” need to be further studies and in larger groups of neonates to validate these approaches. The relatively low numbers of neonates with HIE highlights the importance of multi-centric studies, to increase the numbers of neonates studied, pool knowledge and decrease the risk of inclusion bias. These considerations should be taken into account in future study designs.

## Author Contributions

MD and FP were responsible for reviewing the articles. MD, KC, and FP were responsible in writing the manuscript and for important intellectual content. All authors contributed to the article and approved the submitted version.

## Conflict of Interest

The authors declare that the research was conducted in the absence of any commercial or financial relationships that could be construed as a potential conflict of interest.

## Abbreviations

LC-MS/MS: liquid chromatograpy-tandem mass spectrometry
HIE: hypoxic ischemic encephalopathy
1H NMR: proton nuclear magnetic resonance
TH: therapeutic hypothermia.
